# CDK1 phosphorylation of TAZ in mitosis inhibits its oncogenic activity

**DOI:** 10.18632/oncotarget.5189

**Published:** 2015-09-07

**Authors:** Lin Zhang, Xingcheng Chen, Seth Stauffer, Shuping Yang, Yuanhong Chen, Jixin Dong

**Affiliations:** ^1^ Department of Radiation Oncology, Qilu Hospital of Shandong University, Jinan, Shandong, P.R. China; ^2^ Eppley Institute for Research in Cancer, Fred & Pamela Buffett Cancer Center, University of Nebraska Medical Center, Omaha, NE, USA; ^3^ Department of Oncology, Shandong Provincial Hospital affiliated with Shandong University, Jinan, Shandong, P.R. China

**Keywords:** TAZ, Hippo pathway, mitotic phosphorylation, CDK1, mitotic defects

## Abstract

The transcriptional co-activator with PDZ-binding motif (TAZ) is a downstream effector of the Hippo tumor suppressor pathway, which plays important roles in cancer and stem cell biology. Hippo signaling inactivates TAZ through phosphorylation (mainly at S89). In the current study, we define a new layer of regulation of TAZ activity that is critical for its oncogenic function. We found that TAZ is phosphorylated *in vitro* and *in vivo* by the mitotic kinase CDK1 at S90, S105, T326, and T346 during the G2/M phase of the cell cycle. Interestingly, mitotic phosphorylation inactivates TAZ oncogenic activity, as the non-phosphorylatable mutant (TAZ-S89A/S90A/S105A/T326A/T346A, TAZ-5A) possesses higher activity in epithelial-mesenchymal transition, anchorage-independent growth, cell migration, and invasion when compared to the TAZ-S89A mutant. Accordingly, TAZ-5A has higher transcriptional activity compared to the TAZ-S89A mutant. Finally, we show that TAZ-S89A or TAZ-5A (to a greater extent) was sufficient to induce spindle and centrosome defects, and chromosome misalignment/missegregation in immortalized epithelial cells. Together, our results reveal a previously unrecognized connection between TAZ oncogenicity and mitotic phospho-regulation.

## INTRODUCTION

TAZ (also called WWTR1-WW domain-containing transcription regulator protein 1) is a transcriptional co-activator that is involved in human cancer and stem cell function [1–4]. TAZ promotes tumor growth and metastasis in several types of cancers, including breast cancer [5–7], colon cancer [8–10], non-small cell lung cancer [11–14] and glioblastoma [15]. Correspondingly, TAZ expression/activity is upregulated in several human malignancies [2, 16] and the TAZ locus is amplified in some triple-negative breast cancer [6] and non-small cell lung cancer tumors [12]. Recent studies showed that the TAZ gene is frequently fused with calmodulin-binding transcription activator 1 (CAMTA1) in epithelioid hemangioendothelioma, although the function of this fusion protein in cancer is still unclear [17, 18]. TAZ also plays an important role in embryonic stem-cell self-renewal [19] and confers stem cell-like properties in breast [6] and oral [20] cancer cells.

TAZ activity/function is regulated largely through the Hippo tumor suppressor pathway, which was originally discovered in *Drosophila* [21] and is highly conserved in mammals [22–24]. The Hippo core kinases large tumor suppressor 1/2 (Lats1/2) phosphorylate and inactivate TAZ by sequestering it in the cytoplasm and promoting ubiquitination-dependent protein degradation [25, 26]. Many cues (e.g. G-protein coupled receptor-Rho GTPase axis, mechanical force and actin cytoskeleton etc.) regulate TAZ activity in a Hippo-dependent manner [2, 4]. Recent work has shown that other signals (e.g. GSK3 or Rho GTPase) can regulate TAZ in a Hippo-independent manner [27, 28]. TAZ also crosstalks with, and is regulated by, Wnt/β-catenin signaling. For example, TAZ, along with β-catenin, is degraded in the absence of Wnt signaling [8] and TAZ (and its paralog YAP) orchestrates the Wnt response by forming a complex with the β-catenin destruction complex [29]. Furthermore, cytoplasmic TAZ (phosphorylated by Hippo) restricts β-catenin nuclear localization/activation directly [30] or through inhibiting Dishevelled phosphorylation [31]. Besides the above regulation, however, it is not known whether and how TAZ is regulated during cell cycle progression/mitosis.

We recently showed that some members of the Hippo pathway are phosphorylated by mitotic kinases Aurora and CDK1 during mitosis [32, 33]. We and others found that TAZ was upshifted on a SDS-polyacrylamide gel (due to phosphorylation) during anti-microtubule drug-induced G2/M arrest [34, 35]; however, the phosphorylation sites and the biological significance of this phosphorylation have remained elusive. In this study, we show that mitotic phosphorylation of TAZ on multiple sites occurs dynamically in cells in a CDK1-dependent manner. Interestingly, mitotic phosphorylation inactivates TAZ's oncogenic activity. Therefore, our data reveal a new layer of regulation for TAZ activity, implicating a link between mitosis and TAZ oncogenicity.

## RESULTS

### TAZ is phosphorylated during anti-mitotic drug-induced G2/M arrest

**Figure 1:**
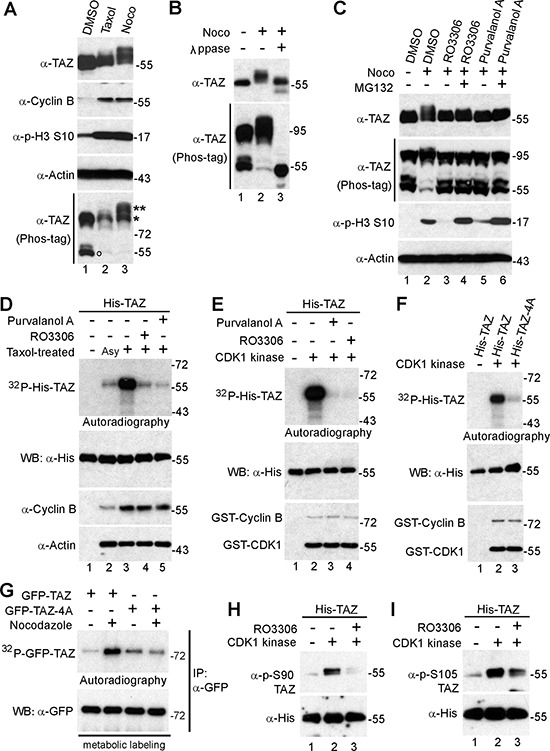
TAZ is phosphorylated by CDK1during G2/M arrest **A.** HeLa cells were treated with DMSO (control), Taxol (0.1 μM for 16 h) or Nocodazole (Noco, 100 ng/ml for 16 h). Total cell lysates were probed with the indicated antibodies. o marks the non-phosphorylated TAZ; * and ** mark the phosphorylated TAZ. **B.** HeLa cells were treated with Nocodazole (Noco) as indicated and cell lysates were further treated with (+) or without (−) λ phosphatase (ppase). Total cell lysates were probed with anti-TAZ antibody. **C.** HeLa cells were treated with Nocodazole (Noco). RO3306 (CDK1 inhibitor) or Purvalanol A (CDK1/2/5 inhibitor) were added (with or without MG132) into the cells 2 h before harvesting the cells. Proteasome inhibitor MG132 was also added (together with CDK1 inhibitors) to prevent cyclin B from degradation and cells from exiting from mitosis. Total cell lysates were subjected to Western blotting with the indicated antibodies. **D.** *In vitro* kinase assays using HeLa cell lysates to phosphorylate recombinant His-TAZ in the presence of ^32^P. Asy: asynchronized; Tax: Taxol-treated. The samples were also probed with cyclin B and β-actin antibodies. **E.** *In vitro* kinase assays with purified CDK1/cyclin B complex. RO3306 (5 μM) or Purvalanol (10 μM) was used to inhibit CDK1 kinase activity. **F.** *In vitro* kinase assays with purified CDK1/cyclin B complex to phosphorylate recombinant His-TAZ or His-TAZ-4A. **G.** GFP-tagged TAZ or –TAZ-4A were transfected into HeLa cells. At 24 h post-transfection, cells were treated with nocodazole (Noco) for 16 h and metabolically labeled in the presence of ^32^P for an additional 2 h as we previously described [33]. **H, I.** *In vitro* kinase assays were done as in (E) except anti-phospho-TAZ S90 and S105 antibodies were used.

We and others showed that TAZ protein is upshifted on SDS-polyacrylamide gels during mitotic arrest induced by Taxol or nocodazole (both agents arrest cells in G2/M by binding to microtubules) [34, 35]. As shown in Figure 1A, the dramatic mobility up-shift of TAZ was readily detected by a Phos-tag gel (Figure 1A). Lambda phosphatase treatment converted all slow-migrating bands to fast-migrating bands, confirming that the mobility shift of TAZ during G2/M is caused by phosphorylation (Figure 1B). TAZ mobility shift/phosphorylation is not likely due to upstream Hippo signaling since the Hippo core is not activated under these conditions [34]. Indeed a very recent study showed that TAZ phosphorylation caused by Taxol treatment is Hippo-independent [36].

Since TAZ is a paralog of YAP and mitotic phosphorylation of YAP is mediated by the mitotic kinase CDK1 [34], we tested whether CDK1 is also responsible for TAZ phosphorylation. As shown in Figure 1C, both RO3306 (a CDK1 inhibitor) and Purvalanol A (an inhibitor for CDK1 and other CDKs) completely reverted the mobility shift of TAZ, suggesting that CDK1 is likely to be responsible for TAZ phosphorylation. Inhibition of other mitotic kinases, specifically Aurora-A, B, C (with VX-680) and PLK1 (with BI2536), did not alter the TAZ phosphorylation (data not shown).

### CDK1 phosphorylates TAZ *in vitro*

Next, we determined whether CDK1 kinase can directly phosphorylate TAZ *in vitro* with His-tagged TAZ as the substrate. Figure 1D shows that Taxol-treated mitotic lysates robustly phosphorylated TAZ and that CDK1 inhibitors greatly reduced phosphorylation of His-TAZ (Figure 1D). Furthermore, purified CDK1/cyclin B complex phosphorylated His-TAZ *in vitro* (Figure 1E). These results indicate that CDK1 phosphorylates TAZ *in vitro*.

There are a total of six sites that fit the proline-directed consensus sequence of CDK1-phosphorylation sites [37]. Two of them (threonine 175 and threonine 285) do not exist in mouse and rat, since they are not conserved, we have excluded them from further study. Interestingly, the remaining four sites (serine 90, serine 105, threonine 326, and threonine 346) in TAZ have been identified as mitotic phosphorylation sites from large scale proteomic studies [38]. Mutating these four sites to non-phosphorylatable alanines (TAZ-4A) almost completely abolished the ^32^P incorporation into TAZ, suggesting that S90, S105, T326 and T346 are the main CDK1 phosphorylation sites (Figure 1F). Metabolic labeling confirmed that wild type TAZ was phosphorylated during Taxol treatment and TAZ-4A was not able to be further phosphorylated during Taxol-induced G2/M arrest (Figure 1G), indicating that these four sites are the main phosphorylation sites during G2/M in cells.

### CDK1/cyclin B complex phosphorylates TAZ at S90 and S105 *in vitro*

We have generated phospho-specific antibodies against S90, S105, T326, and T346. Using these antibodies, we demonstrated that CDK1 phosphorylates TAZ at S90 and S105 *in vitro* (Figure 1H, 1I). Addition of RO3306 abolished the phosphorylation (Figure 1H, 1I). We could not detect a signal when anti-p-TAZ T326 and T346 antibodies were used with these conditions (data not shown).

### Phosphorylation of TAZ occurs in cells during normal mitosis

**Figure 2:**
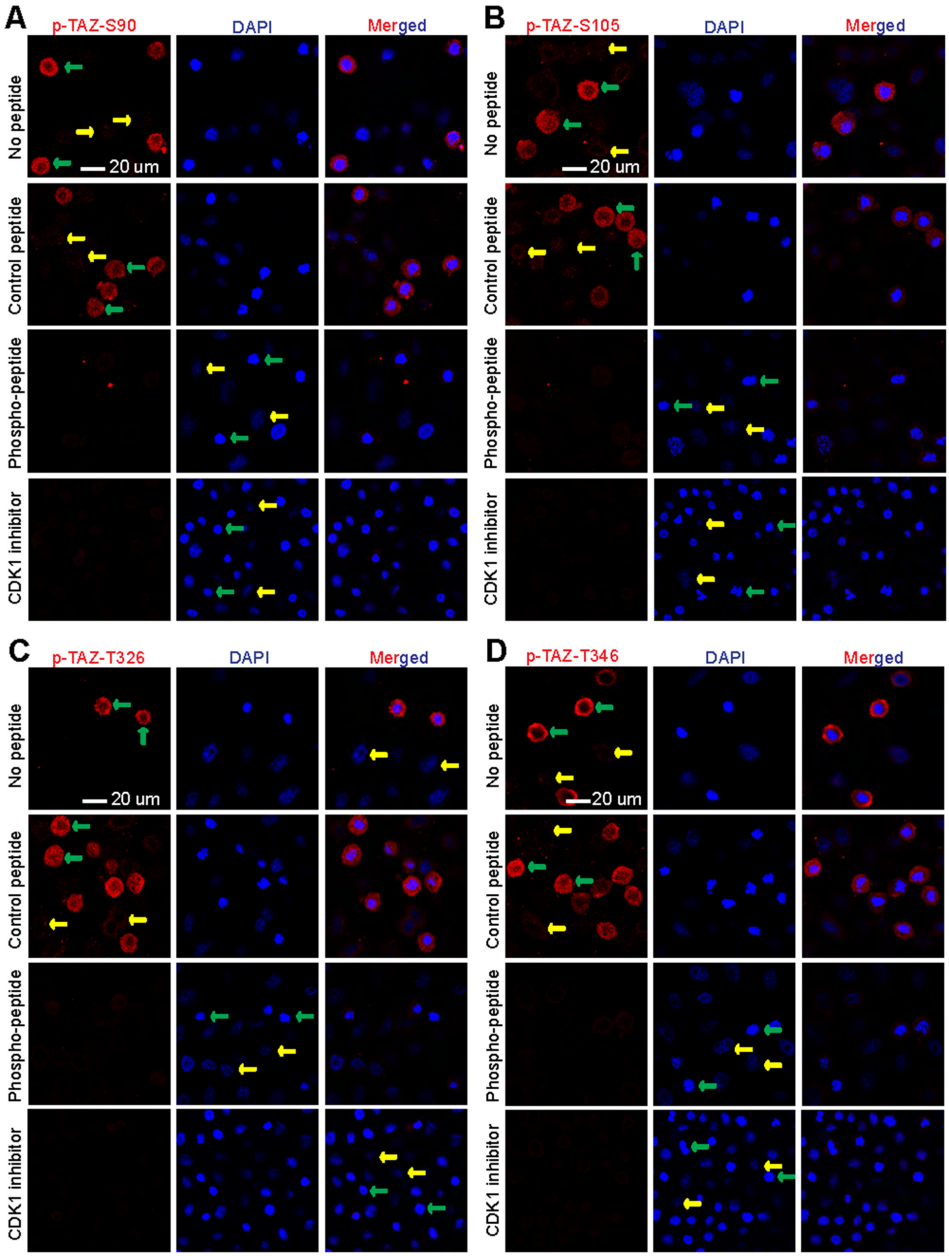
TAZ is phosphorylated at multiple sites by CDK1 during nocodazole-arrested G2/M phase **A.** HeLa cells were treated with nocodazole overnight. The cells were then incubated with or without peptides used for immunizing rabbits prior to phospho-TAZ S90 staining. CDK1 inhibitor (RO3306) was added 2 h before the cells were fixed (bottom panels). **B–D.** Similar experiments were done as in (A) with different phospho-specific antibodies. Green and yellow arrows mark some of the prometaphase cells and the interphase cells, respectively.

Next, we performed immunofluoresence microscopy with these phospho-specific antibodies. Strong and specific signals were detected in nocodazole-arrested prometaphase cells for all antibodies against S90, S105, T326, and T346 (Figure 2A–2D, top panels, green arrows). Very weak or no signal was detected in interphase cells (Figure 2A–2D, yellow arrows). Importantly, phosphopeptide-, but not non-phosphopeptide- (control peptide), incubation largely blocked the signal, suggesting that these antibodies specifically recognize phosphorylated TAZ (Figure 2A–2D, middle panels). Addition of RO3306 largely abolished the signals detected by p-TAZ S90, S105, T326, and T346 antibodies in prometaphase cells, further indicating that the phosphorylation is CDK1 dependent (Figure 2A–2D, low panels).

**Figure 3:**
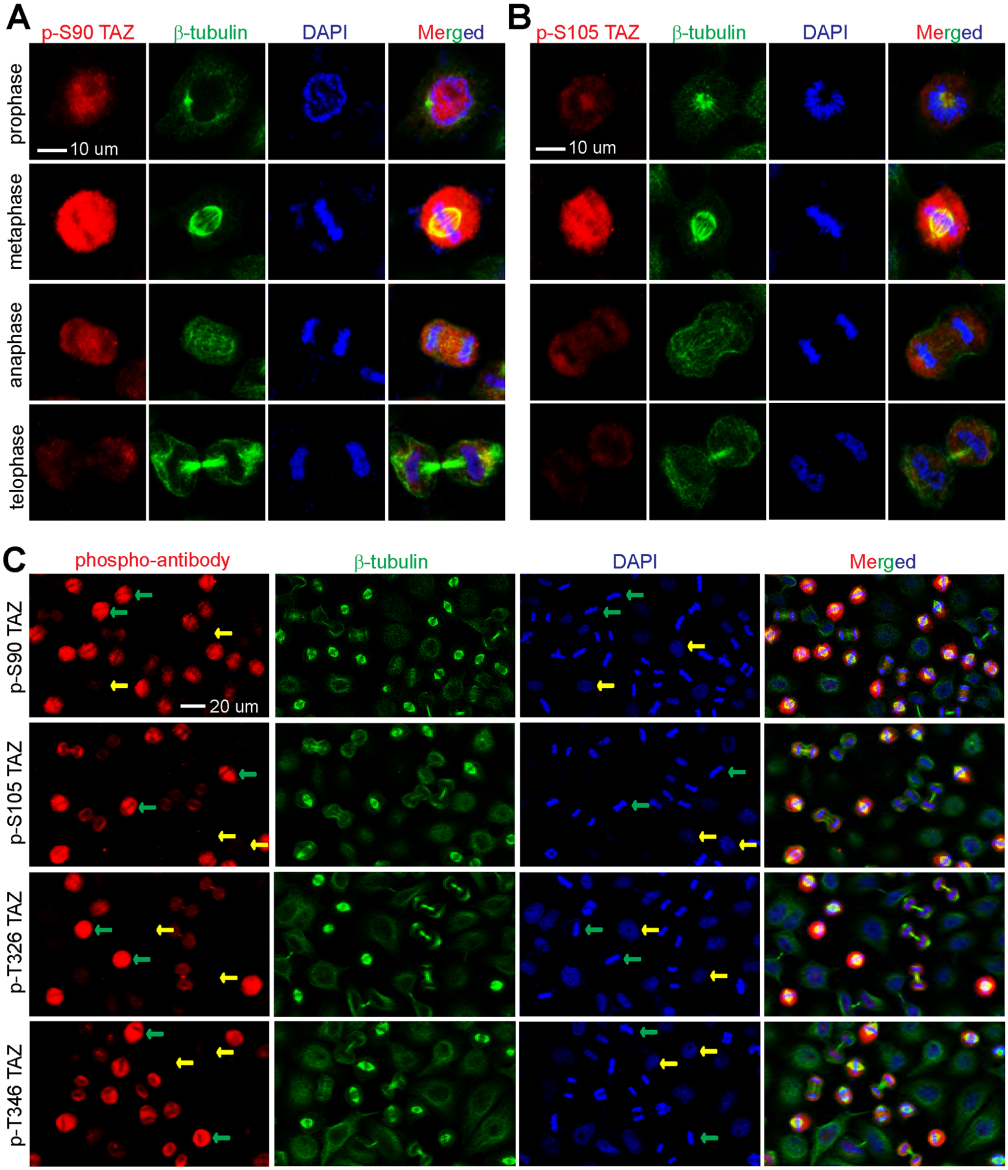
TAZ is phosphorylated at S90, S109, T326 and T346 during normal mitosis **A, B.** HeLa cells were synchronized by a double thymidine (DT) block and release method. Cells were stained with p-TAZ S90 (A) and p-TAZ S105. (B) Cells were co-stained with DAPI and β-tubulin to indicate the various phases. **C.** HeLa cells were synchronized as in (A) and stained with DAPI, phospho-specific antibodies against TAZ, and β-tubulin. A lower power (40X) objective lens was used for photography to view various phases of the cells in a field. Green and yellow arrows in (C) mark the mitotic and interphase cells, respectively.

To further investigate the dynamics of TAZ phosphorylation in cells during unperturbed/normal mitosis, we utilized a double thymidine block and release [39] and determined the phospho-status of TAZ during different cell-cycle phases. We found that the p-TAZ S90 signal was readily detectable in prophase and peaked in prometaphase/metaphase. The signal was then weakened in anaphase and further diminished in telophase and cytokinesis (Figure 3A). We observed similar staining patterns when the p-TAZ S105, T326, and T346 antibodies were used for staining (Figure 3B, 3C and data not shown). These data strongly indicate that mitotic phosphorylation of TAZ occurs dynamically in cells.

### Mitotic phosphorylation inhibits TAZ in EMT and cellular transformation

**Figure 4:**
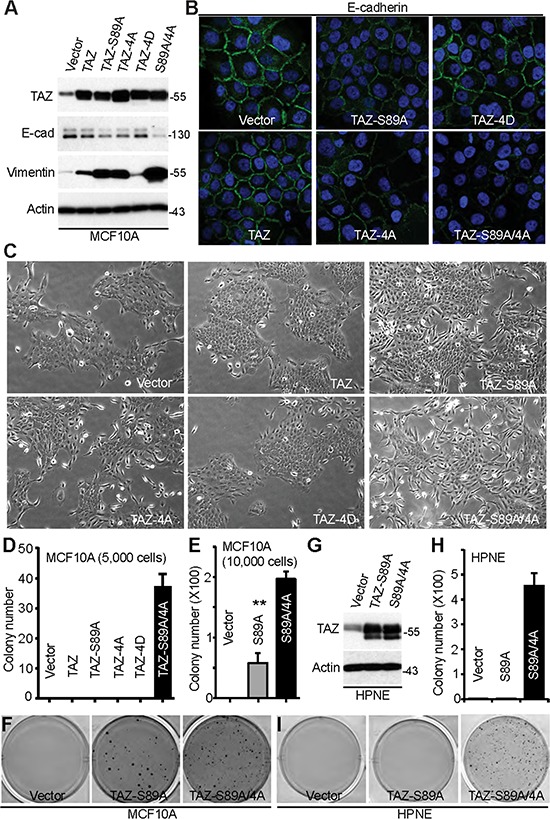
Mitotic phosphorylation of TAZ inhibits EMT and anchorage-independent growth **A.** Establishment of MCF10A cells stably expressing vector, TAZ, TAZ-S89A, TAZ-4A, TAZ-4D, or TAZ-S89A/4A (TAZ-5A). 4A: S90A/S105A/T326A/T346A; 5A: S89A/4A; 4D: S90D/S105D/T326D/T346D. The total cell lysates were probed with the indicated antibodies. **B.** Immunofluorescence staining with E-cadherin in MCF10A cells established in (A). **C.** Morphology change of MCF10A cells expressing vector or various TAZ mutants. **D–F.** Colony assays in soft agar (anchorage-independent growth) in MCF10A cells established in (A). **G.** Establishment of HPNE cells stably expressing vector, TAZ-S89A, or TAZ-5A (S89A/4A). **H, I.** Colony assays in HPNE cells established in (G).

Overexpression of TAZ promotes epithelial-mesenchymal transition (EMT) and transforms MCF10A cells [5, 25, 40], and so we examined the impact of mitotic phosphorylation on EMT, using the TAZ mutants. To do this, we first established pooled cell lines stably expressing TAZ or TAZ mutants (Figure 4A). We confirmed that the epithelial marker E-cadherin was downregulated and vimentin (a mesenchymal marker) was greatly upregulated in cells expressing active TAZ (TAZ-S89A) (Figure 4A). Interestingly, TAZ-4A (non-mitotic phosphorylatable mutant) possesses higher activity in inducing EMT in MCF10A cells when compared to wild type TAZ (Figure 4A, 4B). In contrast, ectopic expression of TAZ-4D (a mitotic phosphomimetic mutant) failed to alter EMT in MCF10A cells (Figure 4A, 4B). Mutating phosphorylation sites to alanines (TAZ-S89A/4A) further increased TAZ-S89A activity in promoting EMT (Figure 4A, 4B), suggesting that mitotic phosphorylation inhibits TAZ in EMT. Consistent with the EMT results, we observed a significant morphology change in MCF10A cells expressing TAZ-4A, but not vector, wild type TAZ or TAZ-4D (Figure 4C). Again, the most significant change was observed in TAZ-S89A/4A-expressing cells (Figure 4A–4C).

MCF10A cells expressing TAZ-S89A/4A formed colonies in soft agar; however, all our other transfectants failed to produce any obvious colonies when 5,000 cells were seeded (Figure 4D). Again, TAZ-S89A/4A possesses higher activity compared to TAZ-S89A in stimulating anchorage-independent growth in soft agar (Figure 4E, 4F). TAZ, TAZ-4A or TAZ-4D overexpression failed to produce colonies in soft agar even when 10, 000 cells were seeded (data not shown). Similarly, only TAZ-S89A/4A-expressing HPNE (an immortalized pancreatic epithelial cell line) cells were able to produce colonies in soft agar (Figure 4G–4I). Together, these data strongly suggest that mitotic phosphorylation inhibits TAZ-mediated cellular transformation in immortalized epithelial cells.

### Mitotic phosphorylation of TAZ impairs cell motility and transcriptional activity

**Figure 5:**
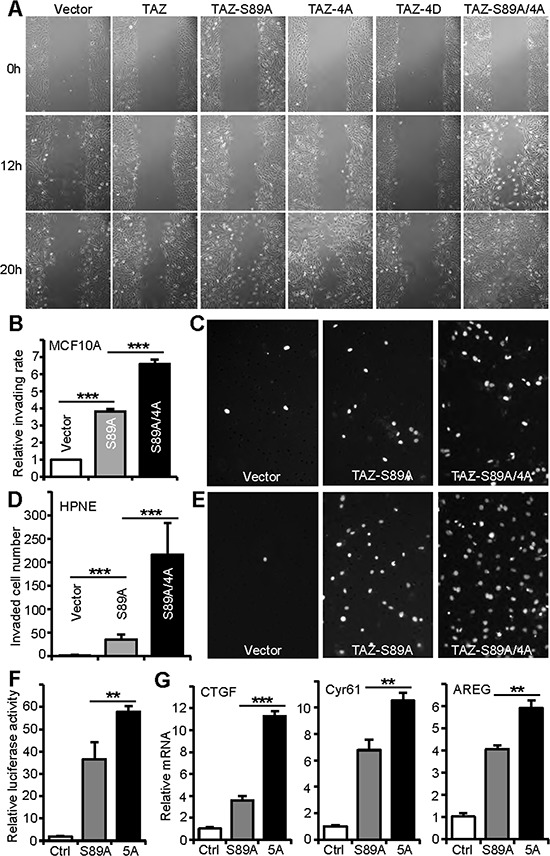
Mitotic phosphorylation of TAZ inhibits its oncogenic and transcriptional activity **A.** Wound healing assays in MCF10A cells expressing various TAZ constructs. **B, C.** Cell invasion assays with MCF10A cells expressing vector, TAZ-S89A or TAZ-S89A/4A. Invading cells were stained with DAPI and representative fields are shown (C). **D, E.** Cell invasion assays with HPNE cells expressing vector, TAZ-S89A or TAZ-S89A/4A. Invaded cells were stained with DAPI and representative fields are shown (E). **F.** Luciferase reporter assays in HEK293T cells. Expression levels of TAZ-S89A and TAZ-S89A/4A are similar in all transfections (data not shown). Ctrl: control (empty vector); 5A: S89A/4A. Data are expressed as the mean ± s.e.m. of three independent experiments (each in triplicate). **p < 0.01 (TAZ5A vs TAZ-S89A) (*t*-test). **G.** Quantitative RT-PCR of YAP targets in MCF10A cells expressing vector, TAZ-S89A or TAZ-S89A/4A. Data are expressed as the mean ± s.e.m. of three independent experiments (in duplicate). ***p < 0.001; **p < 0.01 (TAZ5A vs TAZ-S89A) (*t*-test).

Several studies showed that TAZ/TAZ-S89A also promotes cell migration, invasion and metastasis in animal [5, 20, 41]. We therefore tested whether mitotic phosphorylation affects TAZ's activity in cell motility. As expected, ectopic expression of TAZ or TAZ-S89A increased migration of MCF10A cells assayed by wound healing (Figure 5A). Mutating CDK1-mediated phosphorylation sites to alanines (TAZ-4A) increased migration to a greater extent when compared to wild type TAZ (Figure 5A). In contrast, cells expressing TAZ-4D possess much lower migratory activity than cells expressing wild type TAZ (Figure 5A). Cells expressing TAZ-S89A/4A migrate the fastest (Figure 5A). We further examined the TAZ activity in invasion using Matrigel. Expression of TAZ-S89A greatly enhanced invasion of both MCF10A (Figure 5B, 5C) and HPNE (Figure 5D, 5E) cells. In line with the observations from Figure 4 and Figure 5A, the non-mitotic phosphorylatable mutant (TAZ-S89A/4A) further increased the invading activity when compared to TAZ-S89A (Figure 5B–5E). Again, TAZ-4D-expressing cells (similar to control cells) possess the lowest activity in invasion (data not shown). Together, these data suggest that mitotic phosphorylation of TAZ inhibits cell motility in immortalized epithelial cells.

TAZ is a transcriptional co-activator, and functions mainly through the TEAD1–4 transcription factors in the Hippo pathway [40, 42, 43]. We determined whether mitotic phosphorylation affects TAZ's transcriptional activity using luciferase reporter assays. As shown in Figure 5F, expression of TAZ-5A (TAZ-S89A/4A) significantly increased the luciferase activity compared with TAZ-S89A (Figure 5F). Expression of TAZ-4D failed to significantly induce TEAD-luciferase activity (data not shown). These results suggest that mitotic phosphorylation impairs TAZ's transcriptional activity. Consistent with these observations, the target genes’ expression was further induced by overexpression of TAZ-5A when compared with TAZ-S89A (Figure 5G). Collectively, these data strongly indicate that mitotic phosphorylation inhibits TAZ's oncogenic activity.

### Non-phosphorylatable (active) TAZ induces mitotic abnormalities

**Figure 6:**
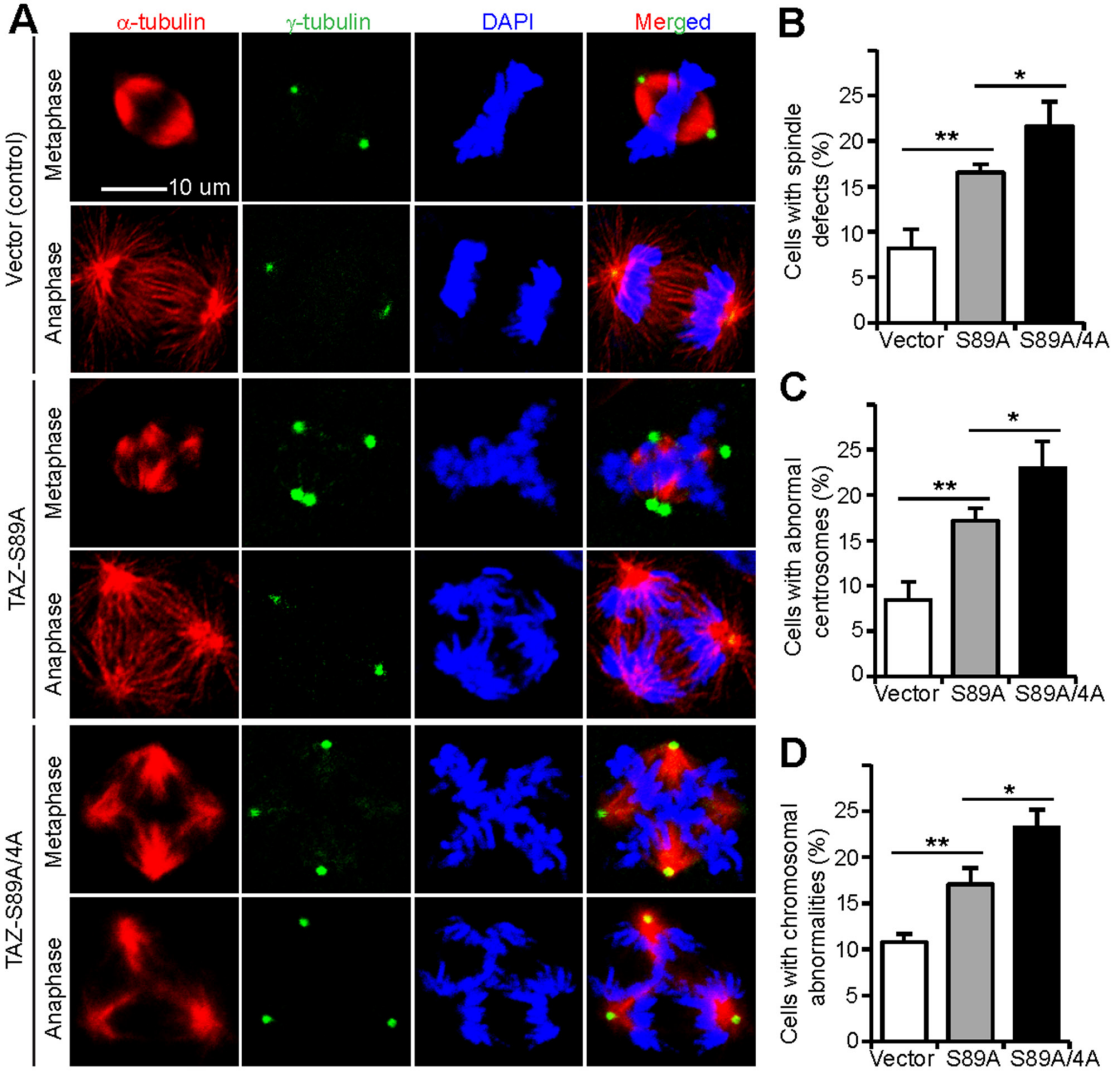
Non-phosphorylatable TAZ induces mitotic defects in MCF10A cells **A.** Representative photos of normal mitosis (vector control) and mitotic abnormalities (TAZ-S89A or TAZ-S89A/4A) in MCF10A cells. MCF10A cells stably expressing vector, TAZ-S89A, and TAZ-S89A/4A (TAZ5A) were established at the same time and maintained at similar passage (around 22–24 at the time of experiments conducted). Cells were stained with α-tubulin antibody, γ-tubulin antibody or DAPI to visualize microtubules (red), centrosomes (green), and chromosomes (blue), respectively. **B–D.** Quantification of mitotic characteristics including microtubule organization/multipolar spindles (B), centrosome number (C), and chromosome alignment (D). Data were collected from *n* = 106, 185, or 243 mitotic cells for vector control, TAZ-S89A, and TAZ-S89A/4A-expressing cells, respectively. Data were expressed as the mean ± s.e.m. of four independent experiments. **p < 0.01; *p < 0.05 (*t*-test).

We next examined whether TAZ or its phosphorylation mutants are able to trigger mitotic defects. MCF10A cells stably expressing vector, TAZ-S89A, or TAZ-5A (TAZ-S89A/4A) were used for this purpose. Consistent with our recent studies, immunofluoresence staining with α-tubulin and γ-tubulin antibodies showed normal microtubule/spindle formation and centrosome number during mitosis in most control cells (Figure 6A). In contrast, mitotic abnormalities (disorganization of microtubules and formation of multipolar spindles) were detected in a significantly higher percentage of cells expressing TAZ-S89A, and to an even greater extent in TAZ-S89A/4A-expressing cells (Figure 6A, 6B). Overexpression of TAZ-S89A or TAZ-S89A/4A also induced abnormal centrosome numbers, as shown by the γ-tubulin staining (Figure 6A, 6C). Not surprisingly, massive chromosome misalignment and chromosome missegregation were observed in a higher percentage of TAZ-S89A- or TAZ-S89A/4A-expressing cells when compared with vector-expressing cells (Figure 6A, 6D). These data suggest that ectopic expression of non-phosphorylatable (active) TAZ is sufficient to trigger mitotic abnormalities in immortalized epithelial cells.

## DISCUSSION

Although recent studies have demonstrated important roles for TAZ in promoting tumorigenesis, the underlying mechanisms are largely unclear. The current study identified novel phosphorylation of TAZ during mitosis and importantly, the mitotic phosphorylation regulates TAZ's oncogenic activity (Figures 4, 5). Interestingly, TAZ-5A (a non-phosphorylatable mutant) drives massive mitotic defects (Figure 6). Thus, TAZ may contribute to cancer development by regulating mitosis-related events, since aberration of mitosis often causes genome instability/aneuploidy and subsequent tumor formation [44].

Our data not only reveal a new layer of regulation for TAZ's oncogenic activity, but also highlight a previously unrecognized mechanism through which TAZ exerts its oncogenic function. Intriguingly, recent studies have shown that most of the Hippo core tumor suppressor proteins, such as Mst1/2, Lats1/2, WW45, Mob1 are involved in regulating mitosis [45–48]. Furthermore, several other regulators of the Hippo pathway, such as Ajuba, Zyxin, KIBRA, as well as the effector YAP, are known to be regulated (phosphorylated) during mitosis and they all play a role in mitotic progression [32–34, 39, 49–53]. Therefore, these studies suggest that the Hippo-YAP/TAZ pathway ensures normal mitosis and that deregulation of the pathway causes mitotic aberrations and tumorigenesis.

Upon treatment with anti-mitotic agents (including Taxol), YAP [34, 35] and KIBRA [32, 33] are phosphorylated by mitotic kinases independently of the Hippo pathway. Another prominent change is the marked increase of Lats2 proteins in response to Taxol treatment [34, 54]. Interestingly, induction of Lats2 and phosphorylation of YAP regulate Taxol sensitivity in cancer cells [35, 54]. Furthermore, TAZ and its downstream targets Cyr61 and CTGF have been shown to be important regulators for Taxol resistance in breast cancer cells [55]. Our current studies show that TAZ is phosphorylated during Taxol treatment and this phosphorylation inhibits its transcriptional activity (Figures 1, 5). Taxol is widely used for treating breast and ovarian cancer patients and drug resistance is one of the major clinical challenges. Therefore, it will be interesting to determine the role of mitotic phosphorylation of TAZ in mediating anti-Taxol drug resistance. Indeed a recent study showed that TAZ is phosphorylated and degraded during Taxol treatment to sensitize cancer cells to Taxol-mediated cell death [36]. These observations suggest that the CDK1-TAZ axis may be druggable for the treatment of anti-mitotic drug resistant cancer patients.

We recently found that YAP (a paralog of TAZ) is required for the spindle checkpoint activation induced by Taxol [49]. YAP regulates the spindle checkpoint through upregulating the spindle checkpoint protein BubR1 in a mitotic phosphorylation-dependent manner [49]. Since the spindle checkpoint is a surveillance mechanism in mitosis [56], these studies suggest that YAP and its mitotic phosphorylation trigger mitotic defects through the dysregulation of the spindle checkpoint machinery. Surprisingly, knockdown of TAZ had no effects on the spindle checkpoint activation and mitotic arrest in the presence of anti-mitotic agents (L.Z. and J.D., unpublished observations), suggesting distinct functions of TAZ and YAP in mitosis. Future studies are needed to address how TAZ and its mitotic phosphorylation are involved in mitosis and how they promote mitotic defects.

Additionally, studies from Yang's group showed that there is also a significant difference between TAZ and YAP phosphorylation induced by Taxol treatment. For example, TAZ is degraded after phosphorylation while YAP protein stability is not affected during Taxol treatment [35, 36]. We found that both YAP and TAZ protein levels are not significantly altered during cell cycle progression when they are dynamically hyperphosphorylated during mitosis [34] (Figure 3, L. Z. and J.D., unpublished observations). Thus, the consequences of mitotic phosphorylation of TAZ differ from that induced by Taxol treatment even though phosphorylation occurs similarly by CDK1 kinase. Furthermore, regarding oncogenic activity, mitotic phosphorylation activates YAP [34] and, in contrast, TAZ's activity is inhibited by mitotic phosphorylation (Figures 4, 5). It is currently not known how TAZ and YAP achieve opposite regulation (negatively and positively, respectively) during mitosis by the same kinase, and so this issue will be explored in our future studies.

## MATERIALS AND METHODS

### Expression constructs

HA-TAZ was a gift from Kun-Liang Guan (Addgene plasmid #32839) [25]. To make the retroviral-mediated and GFP-tagged TAZ expression constructs, the above cDNA was cloned into MaRX™IV vector [57] and pEGFP-C1 vector (Clontech), respectively. Point mutations were generated by the QuikChange Site-Directed PCR mutagenesis kit (Stratagene) and verified by sequencing.

### Cell culture and transfection

HEK293T, HeLa, and MCF10A cell lines were purchased from American Type Culture Collection (ATCC). The cell lines were authenticated at ATCC and were used at low (<20) passages. MCF-10A cells were cultured as described [7, 58]. The immortalized human pancreatic epithelial (HPNE) cells were cultured as we previously described [7]. HEK293T and HeLa cell lines were maintained in DMEM media supplemented with 10% FBS (Hyclone). Attractene (Qiagen) was used for transient overexpression transfections, following the manufacturer's instructions. Nocodazole (100 ng/ml for 16–20 h) and Taxol (0.1 μM for 16 h) were used to arrest cells in G2/M phase unless otherwise indicated. RO-3306 (CDK1 inhibitor) and roscovitine (CDK1/2/5 inhibitor) were from ENZO Life Sciences. Purvalanol A (CDK1/2/5 inhibitor) was purchased from Selleck. All other chemicals were either from Sigma or Thermo Fisher.

### Luciferase reporter assay

Luciferase reporter assays were performed in 24-well plates, using HEK293T cells. 8XGTIIC-Luciferase (Addgene #34615, [59]), SV40-Renilla (Addgene #27163, [60]) and various TAZ mutants were co-transfected in triplicate as we have described previously [7]. Luciferase activity was assayed at 48 hours post-transfection by the Dual-Luciferase Reporter Assay System (Promega) following the manufacturer's instructions.

### Recombinant protein purification

To make His-tagged human TAZ, a full-length TAZ cDNA was subcloned into the pET-21c vector (Novagen/EMD Chemicals). The His-tagged proteins were expressed in E. coli and purified on HisPur™ Cobalt spin columns (Pierce) following the manufacturer's instructions.

### *In vitro* kinase assay

About 1 μg of His-TAZ was incubated with 100 ng recombinant CDK1/cyclin B complex (SignalChem) or HeLa cell total lysates in kinase buffer [32] in the presence of 10 μCi γ-^32^P-ATP (3000 Ci/mmol, PerkinElmer). The samples were resolved by SDS-PAGE, transferred onto PVDF (Millipore) and visualized by autoradiography followed by Western blotting or detected by phospho-specific antibodies.

### Antibodies

The TAZ (V386) antibody from Cell Signaling Technology was used for Western blotting throughout the study. Rabbit polyclonal phospho-specific antibodies against TAZ S90, S105, T326, and T346 were generated and purified by AbMart. Anti-β-actin, anti-GFP, and anti-cyclin B antibodies were from Santa Cruz Biotechnology. Anti-glutathione S-transferase (GST) and anti-His antibodies were from Bethyl Laboratories. Anti-phospho-S10 H3 and anti-vimentin antibodies were from Cell Signaling Technology. Anti-E-cadherin antibody was from BD Biosciences. Anti-β-tubulin (Sigma), anti-α-tubulin (Abcam), and anti-γ-tubulin (Biolegend) antibodies were used for immunofluorescence staining.

### Immunoprecipitation, western blot analysis, metabolic labeling, and lambda phosphatase treatment

Immunoprecipitation, Western blotting, and lambda phosphatase treatment were done as previously described [32]. Metabolic labeling was done as described [33]. Phos-tag™ was purchased from Wako Pure Chemical Industries, Ltd. (cat#: 304–93521) and was used at 20 μM in 8% SDS-acrylamide gels following the manufacturer's instructions.

### Immunofluorescence staining and confocal microscopy

Cell fixation, permeabilization, fluorescence staining, and microscopy were done as previously described [39]. For peptide blocking, a protocol from the Abcam web site was used as we previously described [34].

### Colony formation, cell migration, and invasion assays

Colony formation assays in soft agar were performed as described [7]. *In vitro* analysis of invasion and migration was assessed using the BioCoat invasion system (BD Biosciences) and Transwell system (Corning), respectively, according to the manufacturer's instructions and as we previously described [34, 61].

### Statistical analysis

Statistical significance was performed using a two-tailed, unpaired Student's *t*-test.
